# Gating mechanisms underlying deactivation slowing by two KCNQ1 atrial fibrillation mutations

**DOI:** 10.1038/srep45911

**Published:** 2017-04-06

**Authors:** Gary Peng, Rene Barro-Soria, Kevin J. Sampson, H. Peter Larsson, Robert S. Kass

**Affiliations:** 1 Department of Pharmacology, Columbia University Medical Center, New York, New York 10032, USA; 2 Department of Physiology and Biophysics, Miller School of Medicine, University of Miami, Miami, Florida 33136, USA

## Abstract

KCNQ1 is a voltage-gated potassium channel that is modulated by the beta-subunit KCNE1 to generate I_Ks_, the slow delayed rectifier current, which plays a critical role in repolarizing the cardiac action potential. Two KCNQ1 gain-of-function mutations that cause a genetic form of atrial fibrillation, S140G and V141M, drastically slow I_Ks_ deactivation. However, the underlying gating alterations remain unknown. Voltage clamp fluorometry (VCF) allows simultaneous measurement of voltage sensor movement and current through the channel pore. Here, we use VCF and kinetic modeling to determine the effects of mutations on channel voltage-dependent gating. We show that in the absence of KCNE1, S140G, but not V141M, directly slows voltage sensor movement, which indirectly slows current deactivation. In the presence of KCNE1, both S140G and V141M slow pore closing and alter voltage sensor-pore coupling, thereby slowing current deactivation. Our results suggest that KCNE1 can mediate changes in pore movement and voltage sensor-pore coupling to slow I_Ks_ deactivation and provide a key step toward developing mechanism-based therapies.

KCNQ1 is the pore-forming alpha subunit of a voltage-gated potassium channel that assembles with the beta-subunit KCNE1 in the heart to generate the I_Ks_ current, which is critical for normal repolarization of the cardiac action potential^1^,^2^. Mutations in I_Ks_ subunits are associated with potentially lethal arrhythmia disorders, including long QT syndrome^3^,^4^, short QT syndrome^5^, and atrial fibrillation^6^. Gain-of-function mutations in I_Ks_ subunits that cause atrial fibrillation increase the repolarizing current during repetitive stimulation and shorten the action potential duration^7^, which is thought to reduce the refractory period and predispose the heart to electrical re-entry.

KCNQ1, the pore-forming subunit of I_Ks_, contains a voltage-sensing domain (VSD) and a pore domain. The VSD spans transmembrane helices S1-S4 and contains net positive charges on S4 that allow it to sense changes in membrane potential. The pore domain spans S5-S6 and contains the selectivity filter that allows for K^+^ permeation. The VSD is coupled to the pore domain through interactions between the S4-S5 linker and the C-terminus of S6 and requires the lipid molecule phosphatidylinositol 4,5-bisphosphate (PIP_2_)^8^,^9^,^10^. While KCNQ1 by itself is able to form a homotetrameric potassium channel that is capable of voltage-dependent gating, it is modulated by KCNE1 to generate the cardiac I_Ks_ current, characterized by sigmoidal activation kinetics. KCNE1 consists of a single transmembrane helix and is thought to have multiple and extensive points of contact with KCNQ1 that reside within both the VSD and the pore domain^11^,^12^,^13^,^14^,^15^,^16^. The effects of KCNE1 on KCNQ1 function include a delay in the onset of activation, an increase in current amplitude, and a depolarizing shift in the current-voltage relationship^1^,^2^. Previous voltage clamp fluorometry (VCF) experiments, which simultaneously measured voltage sensor movement and current through the channel pore, have shown that KCNE1 alters the VSD-pore coupling in KCNQ1, slows the opening of the activation gate, and causes a hyperpolarizing shift in the voltage dependence of the main voltage sensor activation and a depolarizing shift in the voltage dependence of current activation^17^,^18^. In addition, it has been shown that voltage sensor activation in KCNQ1 proceeds through an intermediate step before reaching full activation^19^. While KCNQ1 alone can open with intermediate voltage sensor activation, in the presence of KCNE1, channels open after full voltage sensor activation^19^. The ratio of KCNE1 to KCNQ1 subunits in I_Ks_ remains controversial, with some evidence suggesting a fixed 2:4 ratio while others suggesting a flexibility in ratio that serves as a mechanism of channel regulation^20^,^21^,^22^,^23^,^24^.

Atrial fibrillation is the most common cardiac arrhythmia and affects more than 3 million adults in the United States^25^. The much rarer, genetic forms of atrial fibrillation have been associated with gain-of-function mutations in potassium channel subunits, such as two adjacent mutations in KCNQ1, S140G^6^ and V141M^26^. Both are located at the extracellular end of the S1 helix of KCNQ1, which is part of the VSD. Previous studies have shown that both mutations drastically slow channel deactivation^27^,^28^, which can cause accumulation of open channels and abnormally increase the repolarizing K^+^ current. Interestingly, the impact of these mutations on channel deactivation kinetics displays distinct dependence on KCNE1. Whereas S140G slows channel deactivation regardless of the presence of KCNE1, V141M only slows deactivation when KCNE1 is present. This result is supported by a homology model of KCNQ1 showing S140 in proximity to a salt bridge interaction within the VSD and V141 in proximity to KCNE1^12^. Furthermore, cysteine crosslinking studies have shown that V141 crosslinks with residues on KCNE1, whereas S140 does not^28^. Nonetheless, the gating mechanisms underlying the slow deactivation conferred by these mutations remain unknown.

In this study, we use VCF to examine the effects of the two mutations on KCNQ1 gating both in the absence (KCNQ1^S140G^/KCNQ1^V141M^) and presence of KCNE1 (I_Ks_^S140G^/I_Ks_^V141M^). We determine whether the slowing in current deactivation observed is caused by a slowing in voltage sensor deactivation. We show that KCNQ1^S140G^ directly slows voltage sensor deactivation which in turn slows current deactivation, whereas KCNQ1^V141M^ has minimal effect on channel gating. On the other hand, both I_Ks_^S140G^ and I_Ks_^V141M^ slow current deactivation drastically, but I_Ks_^V141M^ does not slow voltage sensor deactivation. Furthermore, I_Ks_^S140G^ slowing of voltage sensor movement is dependent on channel opening, indicating that alternative mechanisms of slowing are present. Interestingly, our kinetic models inform that both I_Ks_^S140G^ and I_Ks_^V141M^ alter VSD-pore coupling and slow pore closing, changing the pathway of deactivation such that channels in intermediate voltage sensor states can either reopen or stay open. These findings provide key insights that aid the development of mechanism-based pharmacologic therapies for arrhythmias associated with KCNQ1 mutations.

## Results

### In the absence of KCNE1, S140G slows voltage sensor and current deactivation, whereas V141M does not

**Figure 1:**
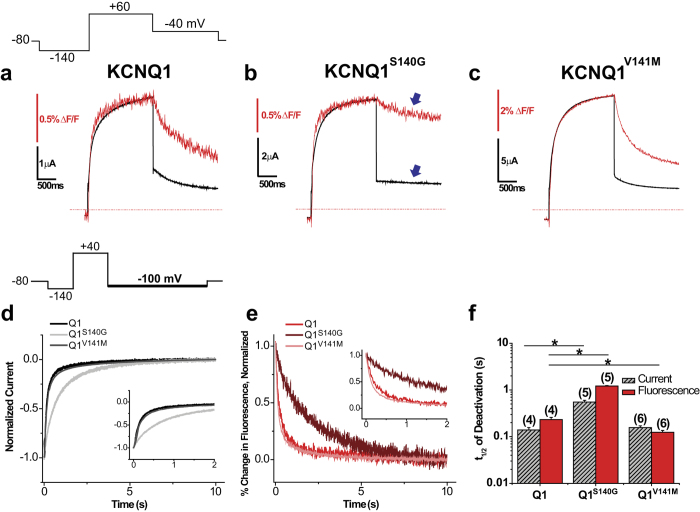
In the absence of KCNE1, S140G slows both current and voltage sensor deactivation, whereas V141M slows neither. (**a**–**c**) Current (black) and fluorescence (red) traces for KCNQ1 (**a**), KCNQ1^S140G^ (**b**), and KCNQ1^V141M^ (**c**) using a single pulse protocol. From a prepulse of −140 mV, a test pulse was applied at +60 mV, followed by a repolarizing step to −40 mV. Cells were held at −80 mV. (**d**) Normalized current during deactivation at −100 mV for KCNQ1, KCNQ1^S140G^ and KCNQ1^V141M^. Inset shows first 2 s of deactivation. Deactivation was examined using the following voltage protocol: from a prepulse of −140 mV, an activating pulse was applied at +40 mV, followed by a repolarizing step to −100 mV. Channels were held at −80 mV. (**e**) Normalized percent change in fluorescence during deactivation at −100 mV. Inset shows first 2 s of deactivation. (**f**) Time to half deactivation (t_1/2_). Data are shown as mean ± SEM (error bars). *P < 0.05.

To study the gating effects of atrial fibrillation mutations using VCF, we employed a KCNQ1 construct containing a cysteine site at extracellular residue G219 that is close to S4 and can be labelled covalently with a fluorophore. This construct has been used in multiple VCF studies^18^,^19^,^29^,^30^, and we refer to it here as KCNQ1. We first used a single pulse protocol to determine whether the atrial fibrillation mutations S140G and V141M affect channel current and voltage sensor movement in the absence of KCNE1. From a holding potential of −80 mV, a prepulse to −140 mV was applied to deactivate voltage sensors to the resting position. Then, a single pulse to +60 mV was applied for 2 s followed by repolarization to −40 mV. In comparison to KCNQ1, both KCNQ1^S140G^ and KCNQ1^V141M^ exhibit similar activation kinetics in current and fluorescence at +60 mV (Fig. 1a–c). However, KCNQ1^S140G^ appears to slow the deactivation kinetics of both current and fluorescence, whereas KCNQ1^V141M^ does not appear to slow either. To determine the voltage dependence of activation, we applied the protocol at multiple voltages ranging from −140 mV to +60 mV and plotted the isochronal activation of fluorescence (FV) and conductance (GV) (Supplementary Fig. S1). Compared with KCNQ1 (FV_1/2_ = −29.3 ± 2.5 mV, GV_1/2_ = −27.2 ± 3.3 mV), KCNQ1^S140G^ (FV_1/2_ = −47.2 ± 2.1 mV, GV_1/2_ = −44.8 ± 2.7 mV) causes a significant hyperpolarizing shift in in both FV and GV, whereas KCNQ1^V141M^ (FV_1/2_ = −23.8 ± 1.3 mV, GV_1/2_ = −36.7 ± 2.0 mV) does not significantly shift either.

The primary pathological effect of these atrial fibrillation mutations is a slowing in current deactivation kinetics. Therefore, in addition to previous single pulse measurements, we further examined deactivation by using a voltage protocol that first activates channels at +40 mV, and then repolarizes to −100 mV in order to deactivate channels more completely. Current and fluorescence were measured simultaneously and time to half deactivation during the −100-mV pulse was determined as a marker of deactivation kinetics (Fig. 1d–f). Compared with KCNQ1 (Ft_1/2_ = 232 ± 27 ms, It_1/2_ = 139 ± 17 ms), KCNQ1^S140G^ (Ft_1/2_ = 1214 ± 27 ms, It_1/2_ = 550 ± 51 ms) significantly slows fluorescence and current deactivation. In contrast, KCNQ1^V141M^ (Ft_1/2_ = 125 ± 11 ms, It_1/2_ = 156 ± 14 ms) slightly speeds fluorescence deactivation without affecting the current deactivation kinetics.

### S140G slows voltage sensor deactivation independently of channel opening

Mechanisms other than a direct slowing in voltage sensor movement may result in an apparent slowing in voltage sensor deactivation. For example, voltage sensor deactivation can be slowed indirectly by slowing of pore closing, since the pore is coupled to the voltage sensor. This concept of “retrograde coupling” has been previously illustrated by Zaydman *et al*. who showed that a pore mutation in KCNQ1 that stabilizes the open channel also stabilizes S4s in their activated states. Upon depleting PIP_2_, which prevents channel opening (presumably by uncoupling the pore from the VSD), the effect on the S4s was abolished^10^. As KCNQ1^S140G^ slows voltage sensor movement and current deactivation by a similar order of magnitude, but with a slightly greater effect on the voltage sensor, we explored whether KCNQ1^S140G^ slows voltage sensor movement when channel pore opening is prevented.

**Figure 2:**
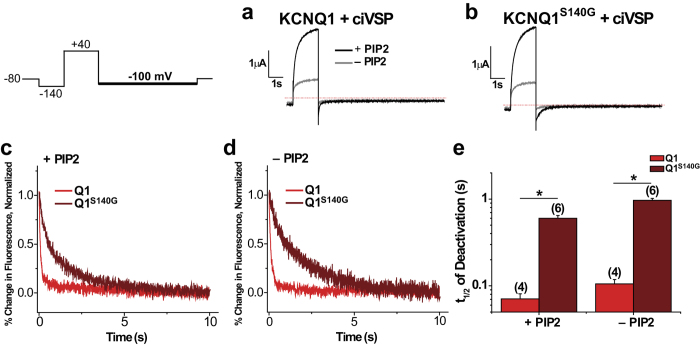
In the absence of KCNE1, S140G slowing of voltage sensor deactivation is independent of channel opening based on PIP_2_ depletion. The following protocol was used: from a prepulse of −140 mV, an activating pulse was applied at +40 mV, followed by a repolarizing step to −100 mV. Channels were held at −80 mV. This protocol was used before and after PIP_2_ depletion by ciVSP, which was activated by repeated depolarization. (**a**,**b**) Current before (+PIP_2_) and after PIP_2_ depletion (−PIP_2_) for KCNQ1 (**a**) and KCNQ1^S140G^ (**b**). (**c**,**d**) Normalized fluorescence deactivation traces at −100 mV for KCNQ1 (red) and KCNQ1^S140G^ (dark red) before (**c**) and after (**d**) PIP_2_ depletion. (**e**) Time to half deactivation (t_1/2_) of fluorescence. Data are shown as mean ± SEM (error bars). *P < 0.05.

Two approaches were used to test whether pore effects indirectly contribute to slowing of voltage sensor deactivation by S140G. In the first approach, either KCNQ1 or KCNQ1^S140G^ was co-expressed with the voltage-sensing phosphatase from *Ciona intestinalis* (ciVSP). Repeated depolarization activates ciVSP, which leads to depletion of PIP_2_ and thus prevents channel opening. A voltage protocol with a pulse to +40 mV followed by repolarization to −100 mV was applied before and after PIP_2_ depletion. We observed a drastic decrease in current amplitude following PIP_2_ depletion in both KCNQ1 and KCNQ1^S140G^ (Fig. 2a,b), indicating a reduction of PIP_2_ levels in the plasma membrane. We then determined the time to half deactivation for fluorescence at −100 mV (Fig. 2c–e). Before PIP_2_ depletion, KCNQ1^S140G^ (Ft_1/2_ = 600 ± 46 ms) slows deactivation kinetics compared with KCNQ1 (Ft_1/2_ = 71 ± 10 ms), as expected. Following PIP_2_ depletion, KCNQ1^S140G^ (Ft_1/2_ = 966 ± 51 ms) still slows the fluorescence deactivation compared with KCNQ1 (Ft_1/2_ = 105 ± 13 ms).

**Figure 3:**
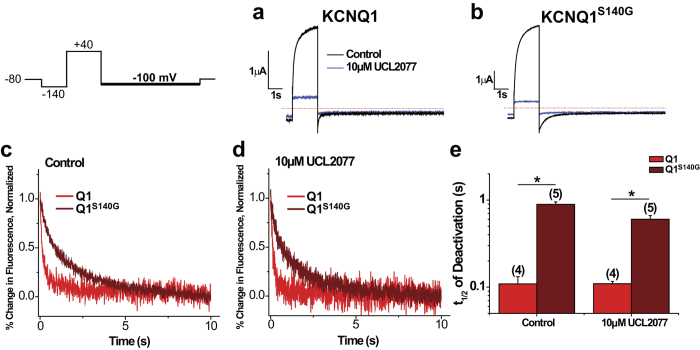
UCL2077 inhibition confirms that KCNQ1^S140G^ slow voltage sensor deactivation independent of channel opening. The following protocol was used: from a prepulse of −140 mV, an activating pulse was applied at +40 mV, followed by a repolarizing step to −100 mV. Channels were held at −80 mV. This protocol was used before and after inhibition of current with 10 μM UCL2077. (**a**,**b**) Current before and after inhibition with UCL2077 for KCNQ1 (**a**) and KCNQ1^S140G^ (**b**). (**c**,**d**) Normalized fluorescence deactivation traces at −100 mV for KCNQ1 (red) and KCNQ1^S140G^ (dark red) in drug-free control (**c**) and in 10 μM UCL2077 (**d**). (**e**) Time to half deactivation (t_1/2_) of fluorescence. Data are shown as mean ± SEM (error bars). *P < 0.05.

In the second approach, we employed UCL2077, an inhibitor of I_Ks_ previously shown to prevent channel opening but still allow S4 movement^18^. Application of 10 μM UCL2077 drastically reduces current of both KCNQ1 and KCNQ1^S140G^ (Fig. 3a,b). When analyzing the kinetics of fluorescence deactivation, we found that both in the control condition and in the presence of 10 μM UCL2077, KCNQ1^S140G^ slows fluorescence deactivation (Control: Ft_1/2_ = 893 ± 59 ms; UCL2077: Ft_1/2_ = 602 ± 61 ms) compared with KCNQ1 (Control: Ft_1/2_ = 109 ± 23 ms; UCL2077: Ft_1/2_ = 109 ± 7 ms) (Fig. 3c–e). Taken together, these experiments show that KCNQ1^S140G^ slows voltage sensor deactivation even in the absence of channel opening.

### In the presence of KCNE1, S140G and V141M cause similar dysfunction in channel currents, but distinct effects on voltage sensor movement

**Figure 4:**
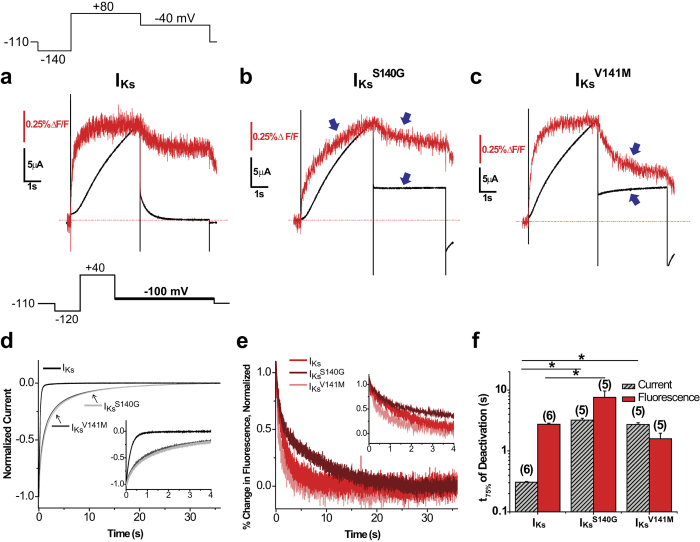
In the presence of KCNE1, S140G slows current deactivation and voltage sensor movement while V141M slows current deactivation without slowing voltage sensor movement. (**a**–**c**) Current and fluorescence traces for I_Ks_ (**a**), I_Ks_^S140G^ (**b**), and I_Ks_^V141M^ (**c**) in the presence of KCNE1 using a single pulse protocol. From a prepulse of −140 mV, a test pulse was applied at +80 mV followed by a repolarizing step to −40 mV. Cells were held at −110 mV. Arrows indicate effect of mutations on current or fluorescence. (**d**) Normalized current during deactivation at −100 mV for I_Ks_, I_Ks_^S140G^, and I_Ks_^V141M^. Inset shows the first 4 s of deactivation. Deactivation was examined using the following voltage protocol: from a prepulse of −120 mV, an activating pulse was applied at +40 mV, followed by a repolarizing step to −100 mV. Channels were held at −110 mV. (**e**) Normalized percent change in fluorescence during deactivation at −100 mV. Inset shows first 4 s of deactivation. (**f**) Time to 75% deactivation (t_75%_). Data are shown as mean ± SEM (error bars). *P < 0.05.

To understand the mechanisms underlying the effects of the mutations on the physiologic I_Ks_ current, we co-expressed KCNQ1 or KCNQ1 mutants with KCNE1 and used VCF to measure voltage sensor movement simultaneously with current. Figure 4a–c shows currents and fluorescence changes from I_Ks_ or mutant channels in response to voltage pulses to +80 mV for 5 s followed by repolarization to −40 mV. For I_Ks_, the fluorescence activation is much faster than the channel’s sigmoidal current activation, as previously reported^18^. Neither mutant channel appreciably affects the kinetics of current activation. However, whereas I_Ks_ deactivates completely at −40 mV, neither I_Ks_^S140G^ nor I_Ks_^V141M^ deactivates appreciably. On the other hand, I_Ks_^S140G^ and I_Ks_^V141M^ exert distinct effects on voltage sensor movement. I_Ks_^S140G^ apparently slows both the activation and deactivation kinetics of voltage sensor movement. On the other hand, I_Ks_^V141M^ does not apparently alter voltage sensor kinetics, but leads to a different steady state level at −40 mV.

As I_Ks_^S140G^ and I_Ks_^V141M^ deactivate incompletely at −40 mV, we applied another voltage protocol in which we activated channels at +40 mV and then deactivated at −100 mV to close channels more fully. We determined the time to 75% deactivation as a marker for deactivation kinetics (Fig. 4d–f). Compared with I_Ks_ (Ft_75%_ = 2.74 ± 0.12 s, It_75%_ = 0.307 ± 0.009 s), I_Ks_^S140G^ (Ft_75%_ = 7.59 ± 2.06 s, It_75%_ = 3.20 ± 0.23 s) significantly slows both voltage sensor and current deactivation by 2.8- and 10.4-fold, respectively. I_Ks_^V141M^ (Ft_75%_ = 1.59 ± 0.37 s, It_75%_ = 2.73 ± 0.18 s) also drastically slows current deactivation kinetics (8.9-fold), but does not slow voltage sensor deactivation. The differing effects of the two mutations on voltage sensor movement when KCNE1 is present suggest that they may slow current deactivation through distinct mechanisms.

### In the presence of KCNE1, S140G slowing of voltage sensor deactivation is dependent on channel opening

**Figure 5:**
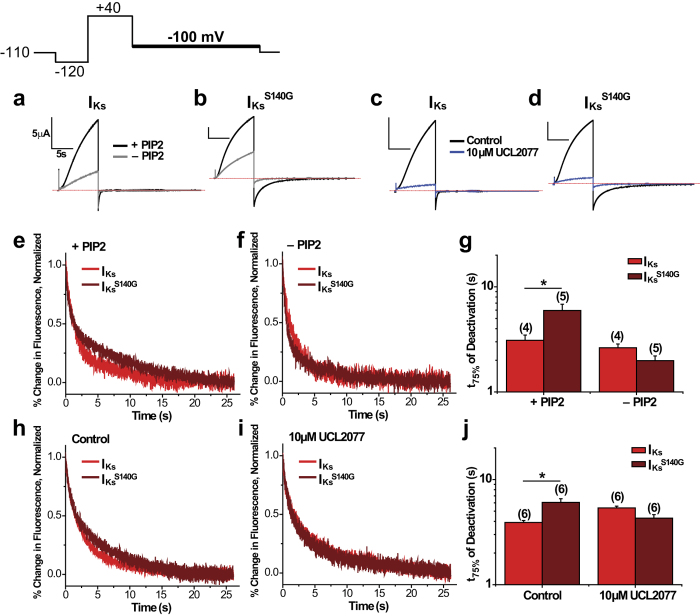
In the presence of KCNE1, S140G slowing of voltage sensor deactivation is dependent on channel opening. The following protocol was used: from a prepulse of −140 mV, an activating pulse was applied at +40 mV, followed by a repolarizing step to −100 mV. Channels were held at −110 mV. (**a**,**b**) Current measured before (+PIP_2_) and after PIP_2_ depletion (−PIP_2_) for I_Ks_ (**a**) and I_Ks_^S140G^ (**b**). (**c**,**d**) Current measured in drug-free control and in 10 μM UCL2077 for I_Ks_ (**c**) and I_Ks_^S140G^ (**d**). (**e**,**f**) Normalized fluorescence deactivation traces at −100 mV before (**e**) and after PIP_2_ depletion (**f**) for I_Ks_ (red) and I_Ks_^S140G^ (dark red). (**g**) Time to 75% deactivation (t_75%_) of fluorescence before and after PIP_2_ depletion. (**h**,**i**) Normalized fluorescence deactivation traces at −100 mV in drug-free control (**h**) and in 10 μM UCL2077 (**i**) for I_Ks_ (red) and I_Ks_^S140G^ (dark red). (**j**) Time to 75% deactivation (t_75%_) of fluorescence in control and in 10 μM UCL2077. Data are shown as mean ± SEM (error bars). *P < 0.05.

We have shown that in the absence of KCNE1, S140G slows voltage sensor deactivation independently of channel opening. To investigate whether the same holds in the presence of KCNE1, we again used PIP_2_ depletion and inhibition with UCL2077 to prevent channel opening. We focused on I_Ks_^S140G^ because I_Ks_^V141M^ does not slow voltage sensor deactivation. When I_Ks_ or I_Ks_^S140G^ was co-expressed with ciVSP, activating ciVSP by repeated membrane depolarization resulted in a reduction of current amplitude in both channels, indicating a reduction in PIP_2_ (Fig. 5a,b). Application of 10 μM UCL2077 also reduces current amplitude in both channels (Fig. 5c–d). Fluorescence deactivation (Fig. 5e–g) was simultaneously measured with current to show that, prior to PIP_2_ depletion, I_Ks_^S140G^ (Ft_75%_ = 5.95 ± 0.88 s) significantly slows fluorescence deactivation compared with I_Ks_ (Ft_75%_ = 3.10 ± 0.38 s). However, following PIP_2_ depletion, I_Ks_^S140G^ (Ft_75%_ = 1.99 ± 0.21 s) no longer slows deactivation compared with I_Ks_ (Ft_75%_ = 2.64 ± 0.22 s), contrary to what we observed in KCNQ1^S140G^. Similarly, in drug-free conditions, I_Ks_^S140G^ (Ft_75%_ = 6.06 ± 0.52 s) slows fluorescence deactivation compared with I_Ks_ (Ft_75%_ = 3.90 ± 0.17 s); however, following current inhibition with 10 μM UCL2077, I_Ks_^S140G^ (Ft_75%_ = 4.30 ± 0.34 s) no longer slows fluorescence deactivation compared with I_Ks_ (Ft_75%_ = 5.36 ± 0.22 s) (Fig. 5h–j). These results show that in the presence of KCNE1, S140G slowing of voltage sensor movement is dependent on channel opening. We additionally measured fluorescence deactivation for I_Ks_^V141M^ under PIP_2_ depletion (Supplementary Fig. S3) as control experiments, since I_Ks_^V141M^ has minimal effects on florescence deactivation. When I_Ks_^V141M^ was co-expressed with ciVSP, repeated membrane depolarization decreased the current amplitude, indicating a reduction in PIP_2_. I_Ks_^V141M^ does not significantly affect fluorescence deactivation kinetics before (Ft_75%_ = 1.79 ± 0.17 s) nor after PIP_2_ depletion (Ft_75%_ = 2.26 ± 0.39 s) compared with I_Ks_. These results were subsequently used to constrain our kinetic model.

### Kinetic modeling informs gating mechanisms for S140G and V141M

**Figure 6:**
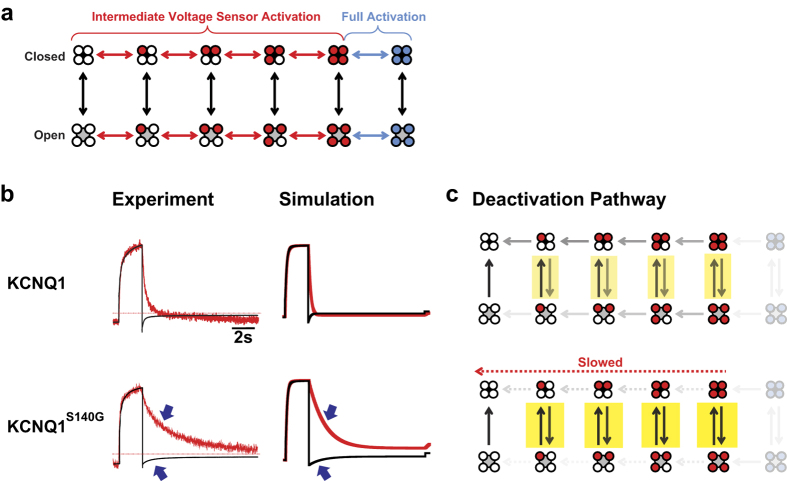
Simulating effects of S140G on KCNQ1 gating in the absence of KCNE1. (**a**) Model of KCNQ1 gating. In this model, KCNQ1 can exist in distinct channel states. Horizontal transitions represent voltage sensor movement, while vertical transitions represent pore opening/closing. Voltage sensors can exist in the resting (white), intermediate (red), or fully activated state (blue). (**b**) Comparing experimental with simulated current (black) and fluorescence (red) traces for KCNQ1 and KCNQ1^S140G^. (**c**) Descriptions of deactivation pathways at −100 mV for KCNQ1 and KCNQ1^S140G^ based on model rates. Arrows with greater opacity represent higher probability of channels entering the transitions specified. In the absence of KCNE1, the probability of channels existing in fully activated voltage sensor states (transparent) is low. The KCNQ1 channels can open and close in intermediate voltage sensor states during deactivation. KCNQ1^S140G^ slows voltage sensor deactivation, which causes increased channel openings and closings, as indicated by highlighting. KCNQ1^S140G^ thus indirectly slows current deactivation.

To further elucidate the mechanisms of the atrial fibrillation mutations on channel gating, we simulated current and fluorescence by modifying a previous kinetic model of KCNQ1 gating^29^, such that voltage sensors that can exist in resting, intermediate, or fully activated states^19^ (Fig. 6a). Voltage sensor movement is represented by horizontal transitions in the model and occurs in two steps: first, independent movement of four VSDs to an intermediate state; second, a concerted movement to a fully activated state. Channel opening can occur in every voltage sensor configuration but increases in probability with each step of voltage sensor activation.

We first obtained a model for KCNQ1 alone (Fig. 6b, top) by fitting model parameters to our data for the kinetics and isochronal activation of both fluorescence and current. We also simulated PIP_2_ depletion by preventing channel opening. In our model, KCNQ1 deactivates through a pathway wherein channels may open or close when some or all voltage sensors are in intermediate states (Fig. 6c, top). As voltage sensor deactivation proceeds, channels become less likely to open. After establishing a working KCNQ1 model, we next modeled KCNQ1^S140G^ by altering only the voltage sensor transitions. We found that by only altering two parameters that determine the rate and the midpoint of the first voltage sensor transition (Supplementary Table S9), all effects of KCNQ1^S140G^ are reproduced: a slowing in current and fluorescence deactivation (Fig. 6b), and a hyperpolarizing shift in isochronal activation of conductance and fluorescence (Supplementary Fig. S4). Slowing of voltage sensor deactivation increases the number of times channels reopen when they are in intermediate voltage sensor states (Fig. 6c, bottom), thereby indirectly slowing current deactivation. Slowing the pore closing transition directly, without slowing voltage sensor movement, cannot account for the effects of S140G on voltage sensor movement (Supplementary Fig. S4). In our model, the slowing of fluorescence deactivation in KCNQ1^S140G^ is independent of channel opening (Supplementary Fig. S4), consistent with our results from the PIP_2_ depletion and UCL2077 inhibition assays.

**Figure 7:**
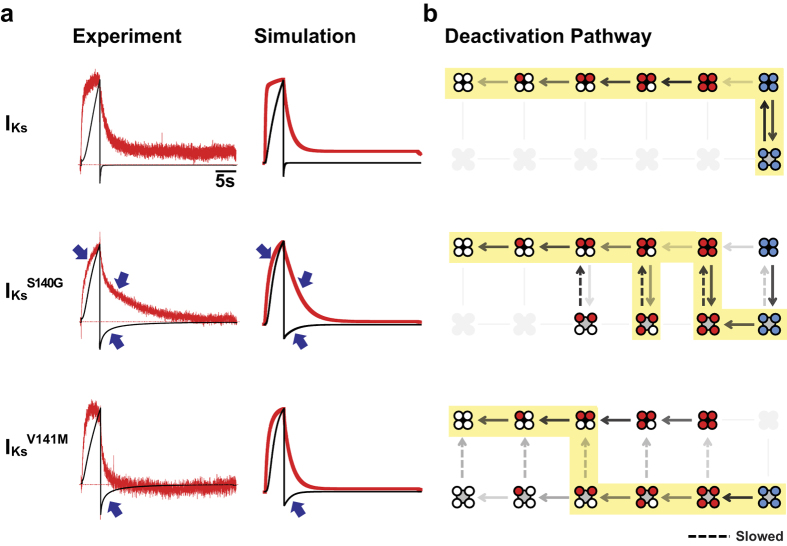
Simulating the gating effects of S140G and V141M in the presence of KCNE1. (**a**) Comparing experimental with simulated current (black) and fluorescence (red) traces for I_Ks_ (top), I_Ks_^S140G^ (middle), and I_Ks_^V141M^ (bottom). (**b**) Description of deactivation pathways at −100 mV based on model rates. Arrows with greater opacity represent higher probability of channels entering the transitions specified. States that channels rarely or never enter are grayed out. Highlighting illustrates the most probable pathway of channel deactivation based on rates at −100 mV, starting from the fully activated open state. I_Ks_ channels opens and closes only when voltage sensors are fully activated (top). I_Ks_^S140G^ alters VSD-pore coupling and slows pore closing, altering the deactivation pathway such that during early steps of voltage sensor deactivation, channels may repeatedly open and close several times (middle). Like I_Ks_^S140G^, I_Ks_^V141M^ also alters VSD-pore coupling to allow channel opening in intermediate VSD states (bottom). In addition, I_Ks_^V141M^ slows pore closing. However, the deactivation pathway is different from I_Ks_^S140G^ in that voltage sensors deactivate to a greater extent prior to pore closing.

We also modeled the effects of S140G and V141M in the presence of KCNE1 using the same gating scheme outlined above. As previously established, KCNE1 alters the coupling between the VSD and the pore in KCNQ1, preventing channel opening when voltage sensors are in the intermediate state^19^. As a result, I_Ks_ follows a deactivation pathway in which pore closing precedes voltage sensor deactivation (Fig. 7b, top). Given these properties of I_Ks_, we modeled the effects of I_Ks_^S140G^ and I_Ks_^V141M^ by varying 4 parameters, with most changes occurring in the rate of the pore closing transition and a factor that controls VSD-pore coupling (Supplementary Table S9). Model parameters were fitted to our experimental data on kinetics, isochronal activation, and PIP_2_ depletion. In addition, as it has been previously shown that the Rb^+^/K^+^ permeability ratio correlates with the degree of voltage sensor activation^19^, we determined the Rb^+^/K^+^ permeability ratios of I_Ks_ and mutant channels to further constrain the model. Using these ratios, we calculated the expected fraction of open channels in the intermediate voltage sensor state following a 5-s activation pulse to +40 mV. All constraining data not summarized in previous sections are presented in the Supplementary section.

Our resulting model simulates the distinct effects of S140G and V141M on current and fluorescence kinetics in the presence of KCNE1. In our model, I_Ks_^S140G^ slows fluorescence and current deactivation, whereas I_Ks_^V141M^ slows current deactivation without slowing fluorescence deactivation (Fig. 7a). These effects are consistent with our experimental data. Furthermore, the model is in agreement with our data on the effects of the two mutations on isochronal activation and fluorescence deactivation under PIP_2_ depletion (Supplementary Fig. S5). The gating effects in I_Ks_^S140G^ are different from those in KCNQ1^S140G^. In contrast to KCNQ1^S140G^, a direct slowing of the voltage sensor movement cannot account for slowing of current deactivation in I_Ks_^S140G^ (Supplementary Fig. S5). Our model of I_Ks_^S140G^ shows that the pathway of channel deactivation is altered, such that channels are allowed to reopen in the intermediate voltage sensor states (Fig. 7b). Interestingly, reopenings delay voltage sensor transitions to indirectly slow voltage sensor deactivation. This is the opposite result from KCNQ1^S140G^, where voltage sensor slowing indirectly slows current deactivation. Our model shows that I_Ks_^V141M^ deactivates through yet a different pathway, where most voltage sensor deactivation occurs prior to pore closing (Fig. 7b). Despite their contrasting deactivation pathways, I_Ks_^S140G^ and I_Ks_^V141M^ share similarities in their gating effects as recapitulated in our models. Both I_Ks_^S140G^ and I_Ks_^V141M^ alter VSD-pore coupling, such that channels in intermediate voltage sensor states are allowed to reopen or remain open. In addition, both I_Ks_^S140G^ and I_Ks_^V141M^ slow the pore closing transition (Fig. 7b), with greater slowing in I_Ks_^V141M^.

## Discussion

**Figure 8:**
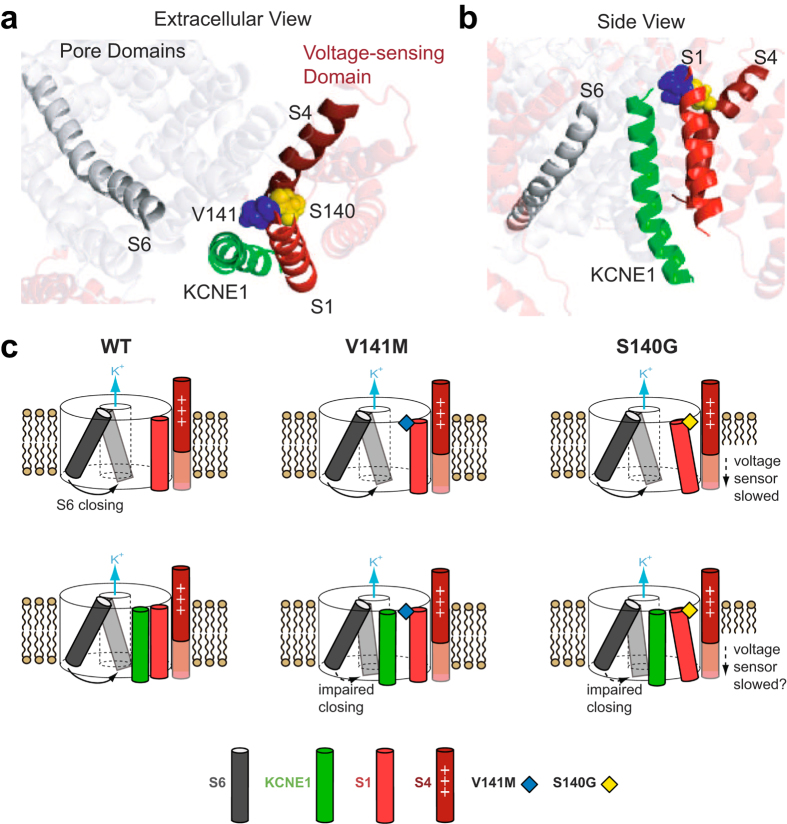
Proposed molecular mechanisms underlying effects of S140G and V141M on channel gating. (**a**,**b**) Homology model of open tetrameric KCNQ1 and KCNE1 from Kang *et al*.^12^ from an extracellular (**a**) or side view (**b**). On the S1 helix, S140 (yellow) points toward S4, whereas V141 (blue) points toward KCNE1. The extracellular end of the S6 helix is in proximity to KCNE1. (**c**) Cartoon representation of proposed molecular mechanisms of S140G and V141M. The channel pore is represented by a cylinder with the K^+^ permeation pathway at its center. For KCNQ1 alone (top row), V141M has minimal effect on channel gating, whereas S140G disrupts S4 and slows its movement. In the presence of KCNE1 (bottom row), both V141M and S140G alter VSD-pore coupling and slow pore closing. V141M may directly disrupt the orientation of KCNE1, impairing motion of the S6 during channel closing. S140G may cause a similar disruption of KCNE1 indirectly through other residues on S1 that face KCNE1.

The slow deactivation caused by two KCNQ1 atrial fibrillation mutations, S140G and V141M, is thought to play a key role in arrhythmogenesis by excessively increasing the repolarizing current of the cardiac action potential during repetitive stimulation. Here we have determined the gating alterations underlying slow deactivation by S140G and V141M both in the absence and presence of KCNE1. Using a combination of VCF and kinetic modeling, we show that in the absence of KCNE1, S140G slows current deactivation indirectly by altering voltage sensor movement. Conversely, a neighboring mutation that also causes atrial fibrillation, V141M, has minimal effect on current and voltage sensor movement in the absence of KCNE1. These results are consistent with prior studies and a published homology model of KCNQ1, in which S140 faces toward the voltage-sensing S4 (Fig. 8a) and is in proximity to a salt bridge interaction between R237 on S4 and E160 on S2 in the open state^27^,^31^. We therefore propose that S140G stabilizes this interaction, thereby explaining the hyperpolarizing shift in the voltage dependence of voltage sensor activation and slowing of voltage sensor deactivation. In contrast, V141 points towards the lipids and away from the rest of the channel, thereby explaining its lack of effect on channel function.

Our findings show that in the presence of KCNE1, the two atrial fibrillation mutations slow current deactivation by altering the gating pathway of deactivation. I_Ks_^S140G^ slows current deactivation by causing channels to reopen during early steps of voltage sensor deactivation. These reopenings delay voltage sensor transitions and indirectly slow voltage sensor deactivation. This is consistent with PIP_2_-dependent slowing of fluorescence deactivation, because PIP_2_ depletion prevents channel opening and therefore removes the reopenings that delay deactivation. Furthermore, closing the channel pore with UCL2077 leads to a similar effect. Taken together, these results demonstrate how the coupling between the channel pore and voltage sensors allows effects on one to induce effects on the other. An alternative explanation for UCL2077’s effect on I_Ks_^S140G^ is a non-specific speeding of fluorescence deactivation by UCL2077, as observed for KCNQ1^S140G^ (Fig. 3e). However, the agreement between our PIP_2_ and UCL2077 results for I_Ks_^S140G^ strengthens the conclusion that slowing of voltage sensor deactivation is dependent on channel opening. The pathway of deactivation for I_Ks_^V141M^ is yet different, in which the pore can close after most or all voltage sensors have deactivated. These two distinct pathways of channel deactivation are consistent with our VCF results and contribute to slowing of current deactivation in the presence of KCNE1.

In the presence of KCNE1, S140G also slows voltage sensor activation. We additionally explored the pore-dependence of this effect using PIP_2_ depletion and UCL2077 inhibition and found that S140G has both pore-dependent and pore-independent effects on I_Ks_ voltage sensor activation (Supplementary Fig. S7). Given the proximity of the S140 residue to the S4^27^,^31^, it is not surprising that S140G has a direct effect on voltage sensor movement. Additional pore-dependent effects would be consistent with a mechanism similar to that of deactivation, where I_Ks_^S140G^ opens and closes in intermediate voltage sensor states, delaying the normal time course of voltage sensor activation.

While the mutations S140G and V141M lead to different pathways of deactivation in the presence of KCNE1, they share two important similarities in their effects on channel gating. First, both mutations alter VSD-pore coupling. It has been previously shown that KCNQ1 alone is able to open with intermediate voltage sensor activation^29^ and that the addition of the beta-subunit KCNE1 results in a channel that only opens after full voltage sensor activation. Our experiments and model show that both I_Ks_^S140G^ and I_Ks_^V141M^ increase open probability of channels in the intermediate voltage sensor states. We propose that the mutations S140G and V141M partially reverse the KCNE1-induced change in VSD-pore coupling, thereby allowing channel opening even when the voltage sensors are in intermediate states. A second similarity between I_Ks_^S140G^ and I_Ks_^V141M^ is a slowing of pore closing. This may be surprising because both S140G and V141M are located near the extracellular end of the S1 helix, which is part of the VSD. However, prior crosslinking studies have shown that the extracellular end of the KCNE1 transmembrane helix is in close proximity to both S1 and S6 of KCNQ1^11^,^14^. Furthermore, the homology model of the KCNQ1-KCNE1 complex from Kang *et al*. shows KCNE1 at a position between the S1 and the S6 of two different KCNQ1 subunits^12^ (Fig. 8b). Functional studies have shown that crosslinking K41 on KCNE1 with I145 on KCNQ1, roughly one turn above V141 on the S1 helix, slows current deactivation ~5-fold at −40 mV^11^. Crosslinking L42 on KCNE1 with V324 on the S6 of KCNQ1 results in extremely slow deactivation^11^. These results suggest that the extracellular end of the S1 helix may interact with KCNE1 to indirectly modulate the S6 helix and influence gate motions. We propose that in the presence of KCNE1, both S140G and V141M slow pore closing through these molecular interactions (Fig. 8c, bottom row). Mutation at V141, a position which also has been shown to crosslink with KCNE1^28^, may directly disrupt the orientation of KCNE1 relative to the S6, restricting molecular motions involved in pore closing. Mutation at S140, which faces toward the VSD of KCNQ1 and does not crosslink with KCNE1^28^, may perturb KCNE1 indirectly through its neighboring residue V141. Although the homology model suggests that S140G should still directly slow voltage sensor deactivation in the presence of KCNE1, it is important to note that KCNE1 itself slows voltage sensor deactivation in our data. If KCNE1 and S140G slow voltage sensor deactivation through a common molecular pathway, no additional effect on VSD deactivation by S140G would be observed in the presence of KCNE1. The possibility of KCNE1 mediating an indirect interaction between S1 and S6, which are part of the VSD and pore domain respectively, may explain how S140G and V141M alter VSD-pore coupling only in the presence of KCNE1. In addition, there are pharmacologic activators of I_Ks_ whose functions depend on the presence of KCNE1^32^. This together with our findings are consistent with the central role of KCNE1 in modulating KCNQ1 gating.

The VCF studies presented here provide insight on the gating mechanisms involved in the slowing of current deactivation by two atrial fibrillation mutations, S140G and V141M. In the absence of KCNE1, S140G slows current deactivation indirectly by slowing voltage sensor deactivation, whereas V141M has minimal effect. In contrast, the slow current deactivation in both I_Ks_^S140G^ and I_Ks_^V141M^ is mainly due to their effects on the voltage sensor-pore coupling and the pore closing transition. This finding is particularly novel, because I_Ks_^S140G^ was previously thought to slow current deactivation primarily by affecting the voltage sensor, similar to KCNQ1^S140G^. Our results hold implications for designing mechanism-based pharmacologic agents for treating arrhythmia associated with mutations in KCNQ1, such as long QT syndrome type 1 and atrial fibrillation. As our study suggests that KCNE1 plays a central role in mediating the slow deactivation by both mutations, future efforts to identify molecular activators and inhibitors of I_Ks_ can benefit from targeting the beta-subunit and inter-subunit interactions in order to alter physiologically relevant channel function.

## Methods

### Molecular Biology

RNA was prepared from DNA sequences in the pGEMHE vector by linearization with NheI, followed by *in vitro* transcription using the mMESSAGE mMACHINE T7 Transcription Kit from ThermoFisher. For DNA sequences in the pSD64TF vector, the restriction enzyme SacI and mMESSAGE mMACHINE SP6 Transcription Kit were used.

A cysteine site was engineered at residue G219 on the extracellular loop of KCNQ1 between S3 and S4 that was to be labelled with a fluorophore. To enhance specificity of labelling, two native cysteine residues (214 and 331) predicted to be accessible to the extracellular environment were mutated to alanine. This construct is referred to as KCNQ1. The terms “KCNQ1^S140G^” and “KCNQ1^V141M^” denote KCNQ1 subunits containing mutations. The terms “I_Ks_^S140G^” and “I_Ks_^V141M^” denote KCNQ1 mutants co-expressed with KCNE1.

### Oocyte Expression

Defolliculated *Xenopus laevis* oocytes were supplied by Ecocyte Bioscience. For KCNQ1 experiments, 50 ng of KCNQ1 RNA was injected into oocytes. For KCNQ1 +KCNE1 experiments, 33 ng KCNQ1 and 10 ng KCNE1 were injected. For PIP2 depletion experiments, 23.5 ng KCNQ1, 7 ng KCNE1, and 14.7 ng ciVSP were injected. For KCNE1 experiments with mutant channels, total RNA injected was reduced, but with same the KCNQ1:KCNE1 RNA ratio. Injection was performed using Nanoject II from Drummond.

### VCF

VCF experiments were performed 2–5 d after injection. Oocytes were labelled with 100 μM Alexa-488 maleimide in ND96 (96 mM NaCl, 2 mM KCl, 1.8 mM CaCl_2_, 1 mM MgCl_2_, 5 mM HEPES, pH 7.5 with NaOH) for 30 min at 12 °C. Following labelling, they were kept on ice to prevent internalization of labelled channels. Oocytes were placed into a recording chamber with animal pole “up” in ND96 solution. 100 μM LaCl_3_ was used to block endogenous hyperpolarization-activated currents. All recordings were performed at 22–24 °C. For UCL2077 experiments, 10 μM UCL2077 in ND96 was pipetted into the recording chamber. 0.1% DMSO was used in drug control experiments.

VCF experiments were performed as previously reported^17^. Isochronal activation was measured for KCNQ1 alone using a protocol with holding potential at −80 mV, a 2-s prepulse to −140 mV, a 2-s test pulse ranging from +60 mV to −140 mV, a 2-s repolarizing pulse to −40 mV, and a sweep interval of 17 s. For KCNQ1 in the presence of KCNE1, isochronal activation was measured using a protocol with a holding potential at −110 mV, a 3-s prepulse to −140 mV, a 5-s test pulse ranging from +80 mV to −160 mV, a 5-s repolarizing pulse to −40 mV, and a sweep interval of 23 s.

### Rb^+^/K^+^ Permeability Ratio

Two-electrode voltage clamp experiments were performed 2–5 d after injection. Oocytes were recorded at room temperature (22–24 °C). Voltage protocol was applied with holding at −110 mV, a 2-s prepulse at −120 mV, a 5-s pulse to +40 mV, followed by repolarization to −60 mV. Currents were recorded under either high external K^+^ (100 mM KCl, 1.8 mM CaCl_2_, 1 mM MgCl_2_, 5 mM HEPES, pH 7.5 with NaOH) or high external Rb^+^ (96 mM RbCl, 4 mM KCl, 1.8 mM CaCl_2_, 1 mM MgCl_2_, 5 mM HEPES, pH 7.5 with NaOH). 100 μM LaCl_3_ was used to block endogenous hyperpolarization-activated currents. Rb^+^/K^+^ permeability ratios were determined using inward tail current measured at −60 mV following the 5-s activation pulse at +40 mV. Inward tail currents were determined following normalization of outward current to calculate Rb^+^/K^+^ ratio. Expected fraction of open channels in the intermediate voltage sensor state at the end of activation was determined for I_Ks_^S140G^ and I_Ks_^V141M^ based on measured Rb^+^/K^+^ ratios.

### Data Analysis

Steady-state voltage dependence of current was determined from exponential fits of tail currents at −40 mV following different test potentials. Fits were extrapolated to beginning of tails to avoid “hooks” that result from channel inactivation. GV relationships were fitted with a Boltzmann equation:


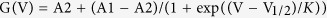


where A1 and A2 are the minimum and maximum, respectively, V_1/2_ is the voltage at half-maximal activation, and K is the slope. Fluorescence was bleach-subtracted and averaged over tens of milliseconds at end of test pulse to reduce errors from signal noise. Voltage dependence of fluorescence was also fitted with a Boltzmann equation.

Statistical data analysis was performed using two-tailed student’s *t-*test or one-way analysis of variance (ANOVA) with Tukey’s post-hoc test. Differences at P < 0.05 were considered significant.

## Additional Information

**How to cite this article**: Peng, G. *et al*. Gating mechanisms underlying deactivation slowing by two KCNQ1 atrial fibrillation mutations. *Sci. Rep.* **7**, 45911; doi: 10.1038/srep45911 (2017).

**Publisher's note:** Springer Nature remains neutral with regard to jurisdictional claims in published maps and institutional affiliations.

## Supplementary Material

Supplementary Information
