# Supplementary material for: Gating mechanisms underlying deactivation slowing by two KCNQ1 atrial fibrillation mutations
Source: Sci Rep. 2017 Apr 6;7:45911. doi: 10.1038/srep45911 (PMC5382920; doi:10.1038/srep45911)
Supplement: Supplementary Information [file srep45911-s1.pdf]

Gating mechanisms underlying deactivation slowing by two KCNQ1 atrial fibrillation mutations – Supplementary File

Gary Peng[1], Rene Barro-Soria[2], Kevin J. Sampson[1], H. Peter Larsson[2]* & Robert S. Kass[1]*

[1]Department of Pharmacology, College of Physicians and Surgeons, Columbia University, New York, New York 10032, USA. [2]Department of Physiology and Biophysics, Miller School of Medicine, University of Miami, Miami, Florida 33136, USA. *Correspondence should be addressed to R.S.K. (email: rsk20@columbia.edu) or H.P.L. (email: PLarsson@med.miami.edu).

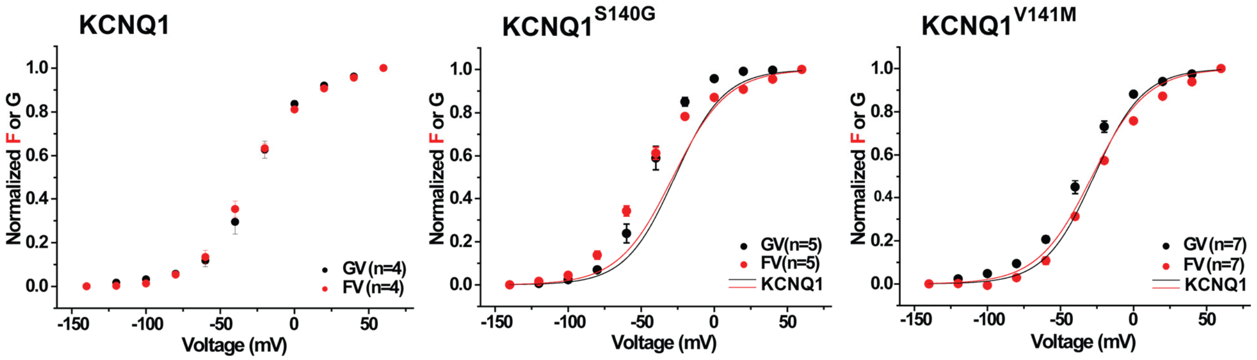

Supplementary Figure S1 – S140G, but not V141M, left-shifts the isochronal activation in the absence of KCNE1. Normalized isochronal (2 s) activation of fluorescence (F) and conductance (G) for KCNQ1, KCNQ1^{S140G}, and KCNQ1^{V141M} are shown. Lines represent KCNQ1 for comparison. Cells were held at -80 mV, prepulsed to -140 mV to deactivate channels, followed by test pulses between +60 mV and -140 mV in intervals of 20 mV.

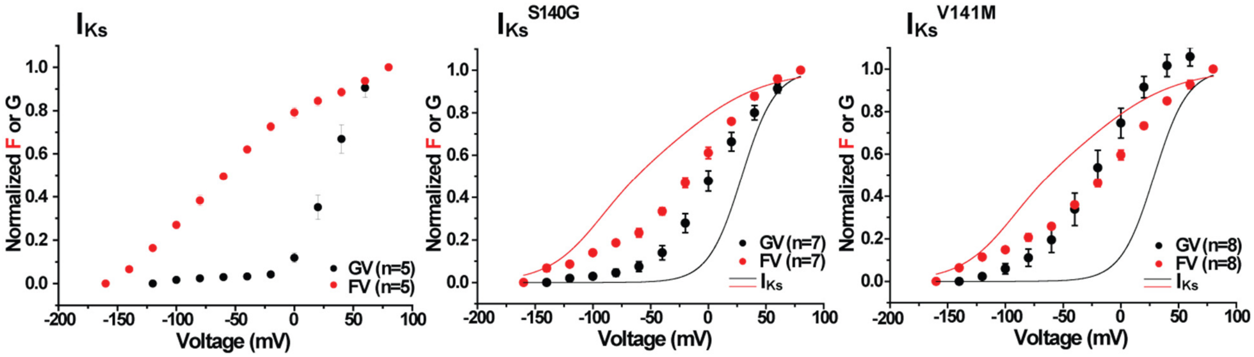

Supplementary Figure S2 - Normalized isochronal (5 s) activation for fluorescence (F) and conductance (G) for I_{Ks}, I_{Ks}^{S140G}, and I_{Ks}^{V141M}. Lines represent I_{Ks} for comparison. Cells were held at -110 mV, prepulsed to -140 mV to deactivate channels, followed by test pulses between +80 mV and -160 mV in intervals of 20 mV.

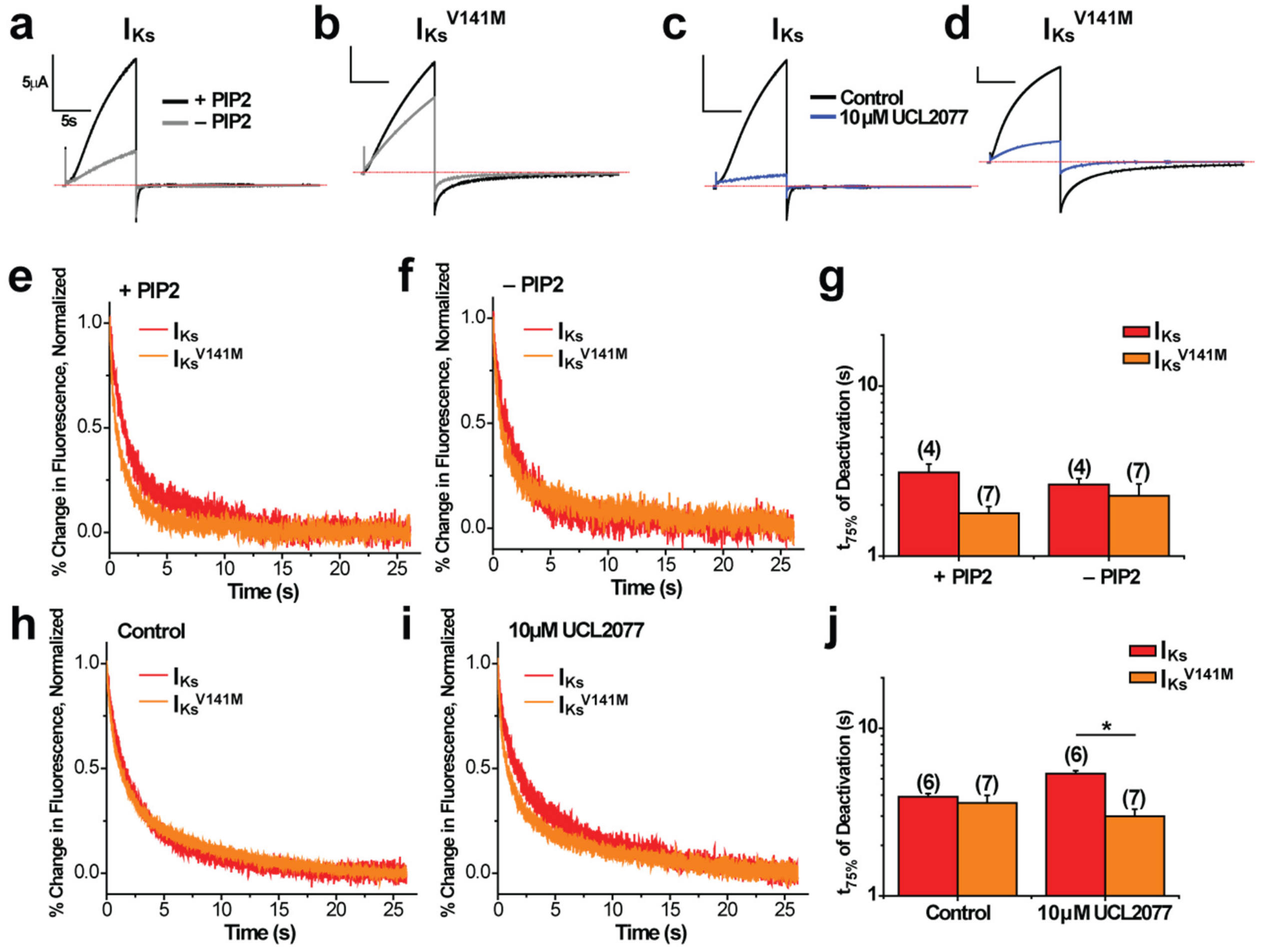

Supplementary Figure S3 – Effect of I_{Ks}^{V141M} on voltage sensor movement either under PIP_2 depletion or UCL2077 inhibition. The following protocol was used: from a prepulse of -140 mV, an activating pulse was applied at +40 mV, followed by a repolarizing step to -100 mV. Channels were held at -110 mV. (a-b) Current measured before (+PIP_2) and after PIP_2 depletion (-PIP_2) for I_{Ks} (a) and I_{Ks}^{V141M} (b). (c-d) Current measured in drug-free control and in 10 µM UCL2077 for I_{Ks} (c) and I_{Ks}^{V141M} (d). (e-f) Normalized fluorescence deactivation traces at -100 mV before (e) and after PIP_2 depletion (f) for I_{Ks} (red) and I_{Ks}^{V141M} (yellow). (g) Time to 75% deactivation ($t_{75\%}$) of fluorescence before and after PIP_2 depletion. (h-i) Normalized fluorescence deactivation traces at -100 mV in drug-free control (h) and 10 µM UCL2077 (i) for I_{Ks} (red) and I_{Ks}^{V141M} (yellow). (j) Time to 75% deactivation ($t_{75\%}$) of fluorescence in control and in 10 µM UCL2077. Data are shown as mean ± SEM (error bars). *$P<0.05$

Simulation

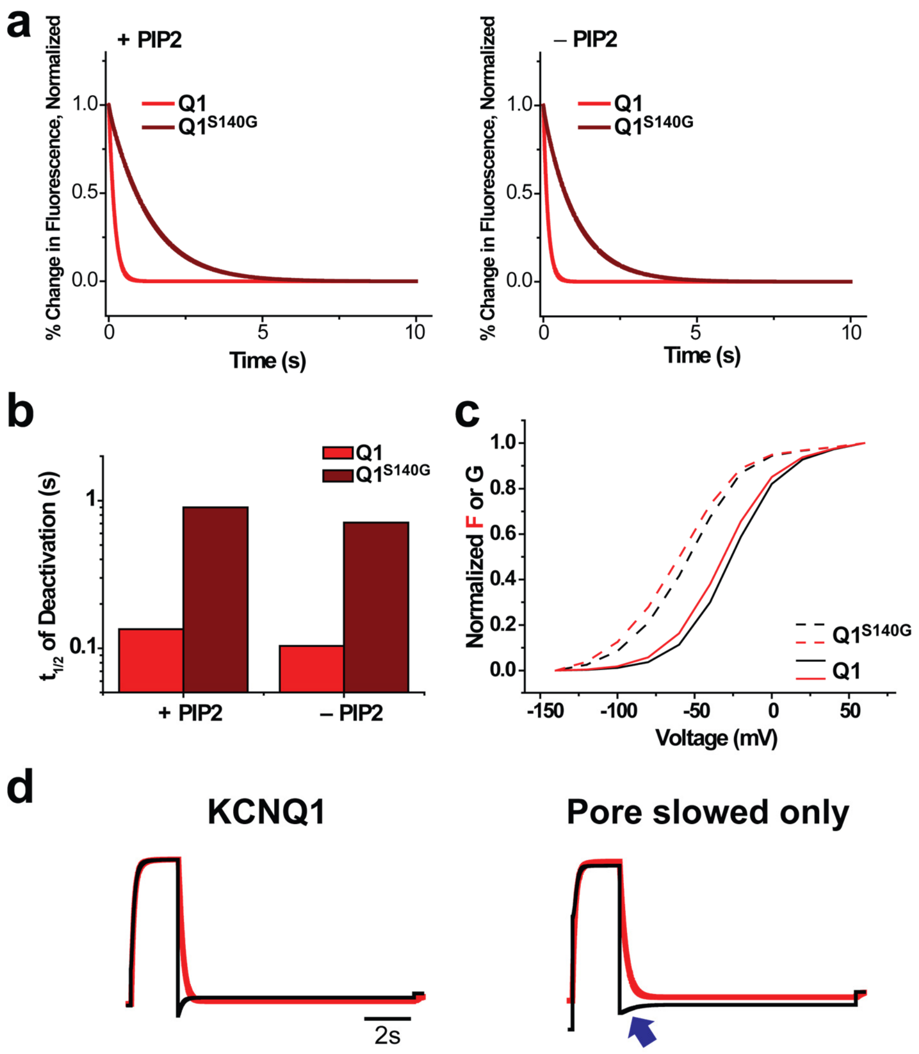

Supplementary Figure S4 – Simulation of KCNQ1 and KCNQ1^{S140G}. (a) Normalized fluorescence deactivation at -100 mV before (left) and after (right) PIP_2 depletion. (b) Time to half deactivation of fluorescence. PIP_2 depletion was simulated by slowing channel opening 10,000 times, effectively preventing channel opening. (c) Normalized isochronal (2 s) activation for conductance (black) and fluorescence (red) for KCNQ1 and KCNQ1^{S140G}. (d) Simulating the effects of a direct pore slowing on current (black) and fluorescence (red) of KCNQ1. Pore slowing does not cause voltage sensor slowing in the absence of KCNE1.

Simulation

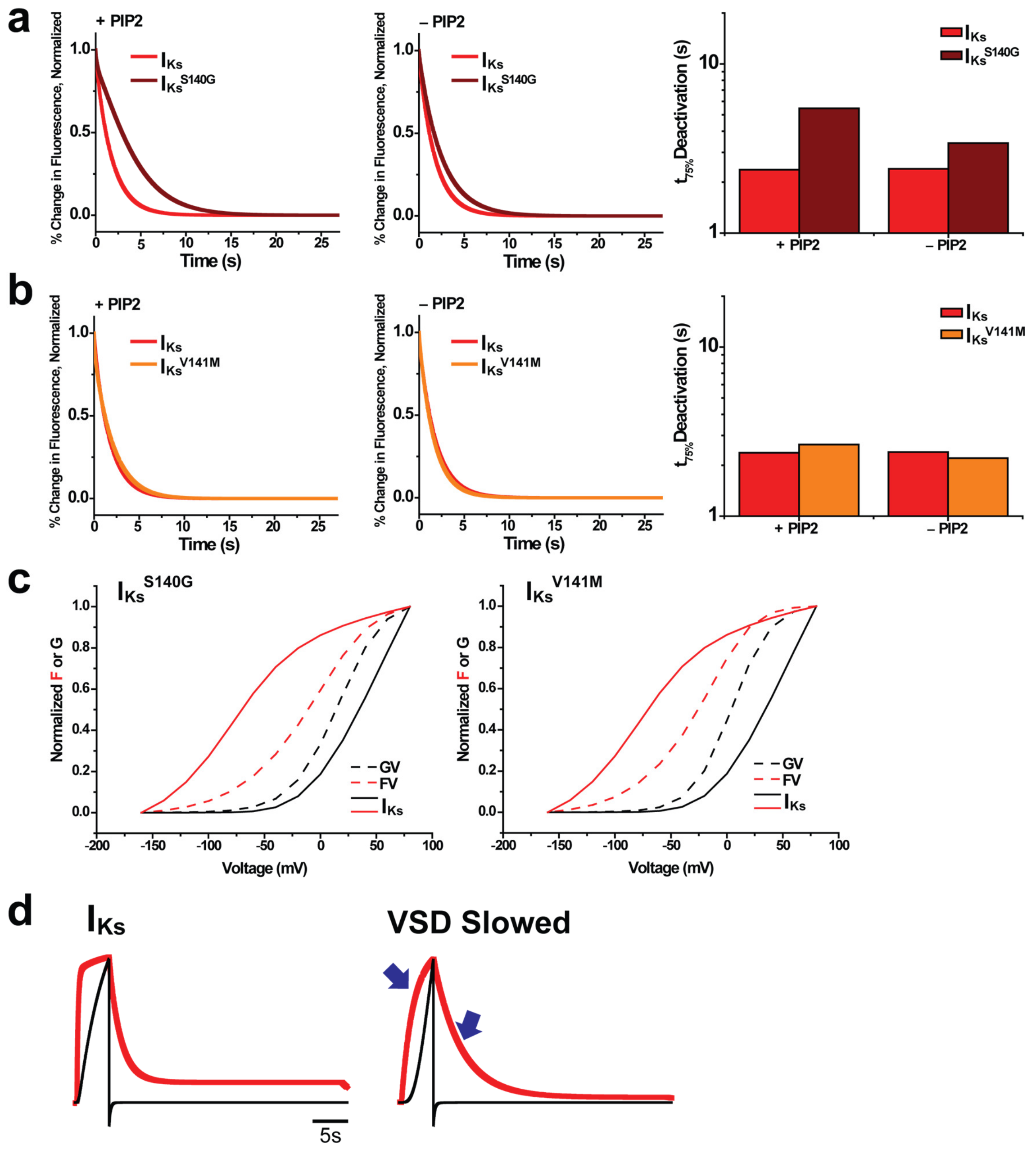

Supplementary Figure S5 – Simulation of I_{Ks} and mutants. (a-b) Simulated normalized fluorescence deactivation traces before (left) and after PIP_2 depletion (middle), and time to 75% deactivation of fluorescence (right) for I_{Ks}^{S140G} (a) and I_{Ks}^{V141M} (b). PIP_2 depletion was simulated by slowing channel opening 10,000 times, effectively preventing channel opening. (c) Normalized isochronal (5 s) activation for conductance (black) and fluorescence (red) for I_{Ks}^{S140G} and I_{Ks}^{V141M}. Lines represent I_{Ks} for comparison. (d) Simulating the effects of a direct slowing of voltage sensor movement on fluorescence (red) and current (black) of I_{Ks}. Voltage sensor slowing does not cause slowing of current deactivation in I_{Ks}.

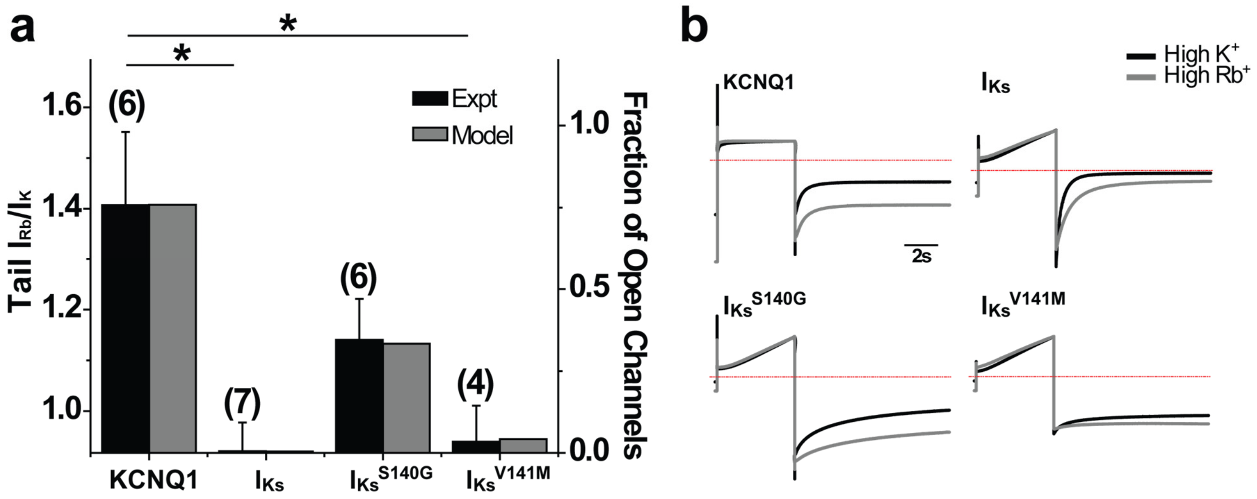

Supplementary Figure S6 – Using Rb^+/K^+ permeability ratio to constrain the kinetic model of I_{Ks} mutants. (a) Comparison of measured tail Rb^+/K^+ ratio with fraction of open channels in the intermediate VSD state following activation, as calculated from model after constraining. Rb^+/K^+ permeability ratio was determined from inward tail current measured either under high external K^+ or Rb^+ concentration. The ratios were converted to expected fraction of open channels in the intermediate VSD state and used to constrain the model. Results for KCNQ1 and I_{Ks} were used for normalization. Data are shown as mean ± SEM (error bars). *P<0.05. (b) Averaged current recordings under high external K^+ or Rb^+ concentration.

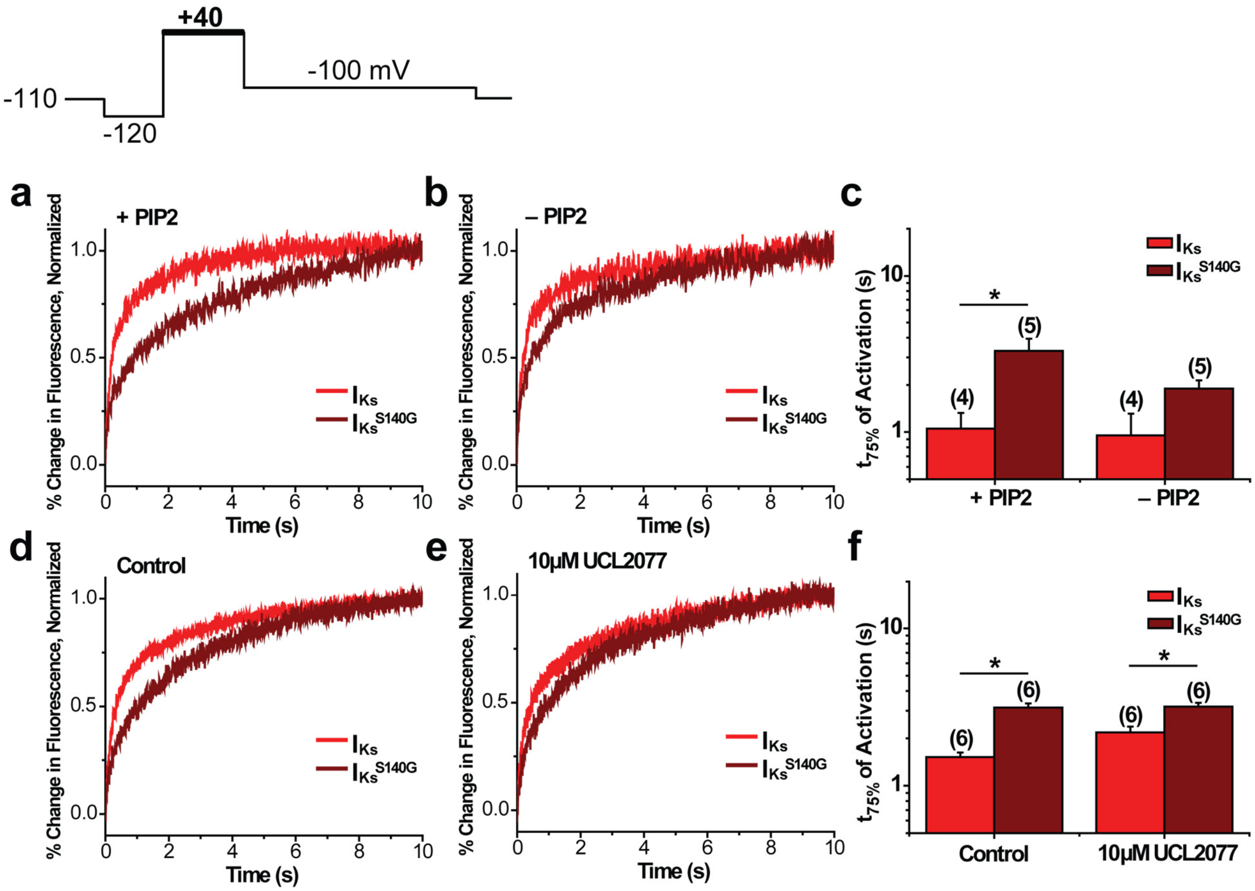

Supplementary Figure S7 – In the presence of KCNE1, S140G slowing of voltage sensor activation is partly dependent on channel opening. The following protocol was used: from a prepulse of -140 mV, an activating pulse was applied at +40 mV, followed by a repolarizing step to -100 mV. Channels were held at -110 mV. (a-b) Normalized fluorescence activation traces at +40 mV before (a) and after PIP_2 depletion (b) for I_{Ks} (red) and I_{Ks}^{S140G} (dark red). (c) Time to 75% activation ($t_{75\%}$) of fluorescence before and after PIP_2 depletion. (d-e) Normalized fluorescence activation traces at +40 mV in drug-free control (d) and in 10 μM UCL2077 (e) for I_{Ks} (red) and I_{Ks}^{S140G} (dark red). (f) Time to 75% deactivation ($t_{75\%}$) of fluorescence in control and in 10 μM UCL2077. Data are shown as mean ± SEM (error bars). *P<0.05.

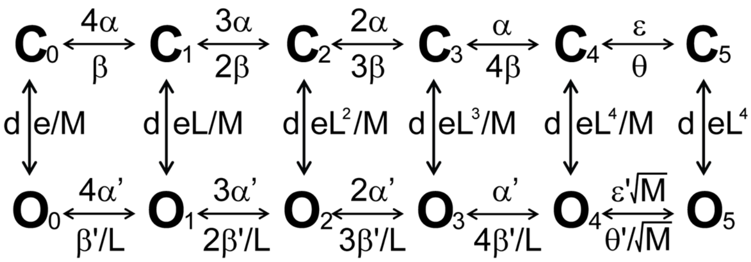

$\alpha = c1 * \exp(c2 * (V_m - V_{half}))$ $\alpha' = c4 * \alpha$

$\beta = c1 * \exp(-c3 * (V_m - V_{half}))$ $\beta' = c4 * \beta$

$\varepsilon = c5 * \exp(c6 * (V_m - V_{half2}))$ $\varepsilon' = c4 * \varepsilon$

$\theta = c5 * \exp(-c7 * (V_m - V_{half2}))$ $\theta' = c4 * \theta$

Supplementary Figure S8 – Model schematic with rates and parameter values.

	**KCNQ1**	**Q1^{S140G}**	**I_{Ks}**	**I_{Ks}^{S140G}**	**I_{Ks}^{V141M}**	**Units**
c1	1.079	0.3624	0.3037	0.1890	0.3037	s^{-1}
c2	0.02977	0.02977	0.02320	0.02320	0.023120	mV^{-1}
c3	0.02700	0.02700	0.008915	0.008915	0.008915	mV^{-1}
c4	0.9659	0.9659	0.9659	0.9659	5	
d	34.03	34.03	26.67	2.036	0.7434	s^{-1}
e	5.533	5.533	0.08381	0.08381	0.08381	s^{-1}
vHalf	-30.56	-70.99	-83.97	-25.68	-32.66	mV
L	1.833	1.833	6.08	6.08	6.08	
c5	1.187	1.187	0.2083	0.2083	0.2083	s^{-1}
c6	0.01413	0.01413	0.01467	0.01467	0.01467	mV^{-1}
c7	0.01354	0.01354	0.02793	0.02793	0.02793	mV^{-1}
vHalf2	98.11	98.11	74.42	74.42	74.42	mV
M	1.399	1.399	1971	21.37	120.0	
fl	0.8559	0.8559	0.8559	0.8559	0.8559	

Supplementary Table S9 – Simulation parameters. Values altered by mutations are highlighted in yellow. The parameter fl represents the fractional fluorescence of the first component of fluorescence.
